# Fellow-Workers and Their Doings

**Published:** 1914-08-29

**Authors:** 


### ROUND THE WORLD.

### Fellow-Workers and their Doings.

[The Editor Invites Early Information and Contributions Marked "Fellow-Workers."]

The four censors for the coming year elected by the Royal College of Physicians are all very well known in the hospital world. Amongst them one finds the Vice-Chancellor of London University, Sir Wilmot Herringham, of "Bart's.," whose work last year, as secretary of the International Congress of Medicine, apart from his valuable publications, must have made him better known to medical men throughout the world than almost any other British physician. Of the others, Dr. Theodore Dyke Acland has been a prominent figure amongst London consultants for many years, and still examines in medicine at Oxford and Cambridge; he is, of course, consulting physician to St. Thomas's and to the Brompton Consumption Hospital.

Dr. Percy Kidd is another well-known adviser of general practitioners in the London area and elsewhere; he is still on the active staff at the London Hospital, and on the consulting staff at the Brompton Consumption Hospital; whilst Dr. Howard Henry Tooth, besides being physician to St. Bartholomew's, is an eminent neurologist, being attached also to the National Hospital for the Paralysed and Epileptic, in Queen Square. He was made a C.M.G. in 1901 for services in South Africa, where he was physician to the Portland Hospital.

Dr. Theodore Shennan, M.D., F.R.C.S.E., who succeeds the late Professor Dean as Regius Professor of Pathology at Aberdeen, is well known to Edinburgh men from his work as pathologist to the Royal Infirmary of the Scottish capital, as well as by his lectures on morbid anatomy in the University there. In addition to research work, Dr. Shennan has written much on the technique of pathological methods; he has also given valuable assistance in the cataloguing of the museum of the Edinburgh Royal College of Surgeons, and was prominent at the Washington International Tuberculosis Congress a few years back. The new member of the Aberdeen faculty pursued his early studies at London and Copenhagen as well as at Edinburgh, and has held several important posts in the course of his career.

The new honorary ophthalmic surgeon to the Bristol Royal Infirmary, Dr. E. H. Edwards Stack, M.B. Cantab., F.R.C.S. Eng., is an old "Bart's" man who settled in Bristol some years ago and became surgeon both to the infirmary and to the Cosham Memorial Hospital. At St. Bartholomew's he was very successful, becoming a Brackenbury (medical) scholar, physician, and ophthalmic house-surgeon. The experience and interest acquired by him in the latter post have steadily borne fruit in the keen attention Dr. Stack has since given to ophthalmic surgery.

Staff-Surgeon Gilbert Francis Syms, who is to be congratulated on winning the Sir Gilbert Blane medal, was formerly a student at Guy's, whence he qualified L.R.C.P., M.R.C.S. in 1908. Subsequently he was house surgeon, clinical assistant, and out-patients' officer there. The Sir Gilbert Blane medal is awarded by the decision of the Royal Colleges of Physicians and Surgeons to the candidate whose name appears first on the list at the examinations for the promotion of naval surgeons to the next grade.

The vacant post of casualty physician to St. Bartholomew's Hospital has fallen to Dr. George Graham, M.D., B.C. Cantab., L.R.C.P., M.R.C.S., who as a student won the senior entrance scholarships of his year at the medical school. Previous to this he won an exhibition at Cambridge, whilst his good work at "Bart.'s" eventually brought him into the ranks of the Otto Beit Memorial Fellows. Dr. Graham has held resident appointments both at St. Bartholomew's and the West London Hospitals; his clinical observations including a study of the alterations of the acid constituents of the gastric juice occurring in cases of stomach cancer.

Congratulations are due to Dr. Charles Jennings Marshall, who has been awarded the Murchison Scholarship, an annual award given by the Royal College of Physicians, and obtained by an examination in clinical medicine held in alternate years at London and Edinburgh. Commencing his medical studies at Cardiff University Medical School, where he won a gold medal, Dr. Marshall later on entered his name as a student at Charing Cross Hospital, and took the L.R.C.P., M.R.C.S. Subsequently he did much useful scientific work as assistant pathologist and bacteriologist there, after which he joined the resident staff as house physician.

An important subject for many elderly people was dealt with at the Aberdeen Congress by Mr. J. S. Fraser—in his paper on the clinical aspects of oto-sclerosis. Giddiness, deafness, and noises in the ear are the bane of many people past middle life, and it appears to have been proved that in many instances these troublesome symptoms are due to sclerotic changes in the middle ear. Although Mr. Fraser, who is assistant surgeon to the ear, throat, and nose department of the Edinburgh Royal Infirmary, and aural surgeon to the Leith Hospital, did not indicate any new method of dealing with this condition, he brought forward a good deal of evidence throwing light on its pathology; and, of course, better understanding of causes holds out further hope of remedy.

The treatment of oto-sclerosis was, indeed, dealt with at the same meeting by Mr. G. J. Jenkins, who expressed the view that massage and other physical measures do more harm than good. Mr. Jenkins, who is assistant aural surgeon to King's College Hospital, and surgeon to the ear, throat, and nose department of the German Hospital, very tersely pointed out the advisability of deaf persons afflicted with oto-sclerosis avoiding alcohol and quacks.

The importance of the after-care of persons who have been treated for consumption can scarcely be over-estimated, as by this means not only may serious relapse be prevented, but the individual may be found suitable occupation and prevented from suffering from that unreasonable fear of infection which still appears to influence many employers and others. An interesting review of after-care schemes was recently put before the National Association for the Prevention of Consumption by Dr. H. W. McConnell, M.A., M.B., M.R.C.S., a member of the Council of that body, who is also hon. secretary to its After-care Committee.

